# Effects of winter and spring housing on growth performance and blood metabolites of Pengbo semi-wool sheep in Tibet

**DOI:** 10.5713/ajas.18.0966

**Published:** 2019-03-07

**Authors:** Yan Mei Jin, Xiao Qing Zhang, Warwick B Badgery, Peng Li, Jun Xi Wu

**Affiliations:** 1 Marine College, Shandong University at Weihai, Weihai 264209, China; 2 Institute of Grassland Research, Chinese Academy of Agricultural Sciences, Hohhot 010010, China; 3 New South Wales Department of Primary Industries, Orange Agricultural Institute, Orange, NSW 2800, Australia; 4 Institute of Geographic Science and Natural Resources Research, Chinese Academy of Sciences, Beijing 100101, China

**Keywords:** Tibet, Cold Stress, Sheep, Winter Housing, Serum Metabolites

## Abstract

**Objective:**

Sixty Pengbo semi-wool sheep ewes (approximately 1.5-years-old; 31.33±0.43 kg) were randomly assigned to two groups, either grazing (G) or dry lot feeding (D), to examine the effects of traditional daily grazing and dry lot feeding on performance and blood metabolites during the cold season in Tibetan Plateau.

**Methods:**

The ewes in the G group were grazed continuously each day and housed in one shed each evening, while the ewes in the D group were housed in another shed all day. All animals were fed 400 g/d of commercial concentrate, and grass hay was available freely throughout the experimental period.

**Results:**

Compared with the G group, the ewes in the D group had higher (p<0.05) live weight and weight gain. The D group ewes had greater (p<0.05) numbers of white blood cells and platelets, while they had lower (p<0.05) platelet-large cell ratios, cholesterol, high-density lipoprotein cholesterol and glutathione peroxidase, as compared with the G group ewes. Additionally, three serum metabolites, abscisic acid, xanthoxin and 3,4-dihydroxy-5-polypren, were upregulated (p<0.05) in the G group in comparison with the D group.

**Conclusion:**

In conclusion, a dry lot feeding regime during the winter and spring period will increase the productivity of sheep and improve blood physiological and biochemical profiles.

## INTRODUCTION

The Xizang (Tibet) autonomous region, located in the southwestern area of China, is the main part of the Tibetan Plateau known as “the roof of the world”, averaging 4,000 m in elevation. The region is of key importance to the ecological security of China and southeastern Asia, and contains some of the most sensitive and fragile ecosystems. Grasslands are the principal ecosystems of the region and the utilization of the forage they produce for animal production is the leading industry. The area of natural grassland in Tibet is 820×l0^6^ ha, accounting for 20.5% of the total grassland area of China, the largest area of grassland in China. In recent decades, excessive utilization and destruction by overgrazing has led to serious degeneration of the grasslands in Tibet. It has been reported that 43 million ha [1], about 26% to 46% of the total grassland area in Tibet, has already been degraded significantly. This situation has detrimentally affected the region’s environment and ecology and its sustainable use for pastoral livestock production.

Sheep are an important livestock species, providing daily necessities, financial income and livelihood for nearly 9.8 million farmers over the entire area of the Xizang-Qinghai Plateau [1]. Livestock productivity is low [2], owing to the serious degeneration of natural grasslands and long-established traditional rearing systems in which farmers focus on animal survival for much of the year with short periods of livestock growth over summer. To improve the ecological environment and livestock production, the “Retire Livestock and Restore Grassland Policy”, introduced by the government, is being implemented, and traditional grazing management is being transitioned to a semi-grazing and semi-housing system, using animal shelters for much of the year. The major problem for pastoral livestock production on the Tibetan grasslands is managing yearlong production systems in a grassland with a short (3- to 4-month) growing season [3] over late spring and summer, when more than 70% of the annual precipitation falls, followed by dry and cold periods from autumn through to early spring. Animals are traditionally grazed all year, but grasslands can only maintain or increase their live weights in summer. In winter, farmers in these regions traditionally take livestock out to graze daily on poor quality pasture. The animals, especially pregnant ewes, are exposed to cold and wind stress that causes body heat to be quickly lost. Feeding indoors in winter is an effective way to alleviate grazing pressure on native grassland and to protect the ecosystem for sustainable utilization of grassland resources. In addition, housing and indoor feeding allows the animals to maintain their body temperature during daily grazing and reduces the extra energy requirement of these animals [4]. This, consequently, reduces weight loss and increases financial incomes during winter and spring [5,6]. Previous studies have focused on animal liveweight changes; however, little is known about physiological status and blood metabolites when animal management is changed from traditional grazing systems to semi-grazing and semi-housing systems.

The aim of the present study was to assess the effects of traditional daily grazing (G) and dry lot feeding (D, no grazing) on the growth performance, blood physiological and biochemical profiles, as well as antioxidant capacity and metabolites in the serum of Pengbo semi-wool sheep ewes during winter–spring in a Tibetan alpine pastoral area.

## MATERIALS AND METHODS

### Study site

The experiment was conducted at a sheep raising cooperative in Linzhou County (91°16 E, 29°53 N; altitude 3,926 m above sea level), Tibet, China, from December 2016 to April 2017. Linzhou County is typical of the “One River and Two Streams region” in Tibet, belonging to a plateau monsoon and semi-arid climate regime, with mean annual temperature and precipitation of approximately 2.4°C to 5.8°C and 440 mm. The main rainfall occurs in summer (between June and August), and daily air temperatures are often below 0°C in winter; in the coldest period (December to January) it can be −15°C at night. The daily range of the surface ground temperature is large, and alternating negative and positive temperatures are frequent, occurring on about 150 to 230 days every year [7]. It is one of the places with the highest number of strong-wind days in China. Generally, the annual average wind speed is 3.0 to 4.0 m/s. The duration for which the wind speed is greater than 17.2 m/s is 30 to 129 days, and 40% to 50% of this occurs in spring [7], which accelerates the lowering of the temperature. The frost-free period is 120 days, from May through September. Grassland below 4,200 m above sea level is dominated by *Pennisetum centrasiaticum* Tzvel. The aboveground biomass of the desirable species is 225 to 300 kg dry matter (DM)/ha in summer and it is here that the yield is very low in winter–spring. The Pengbo semi-wool sheep is the major source of income for local farmers. These sheep typically produce 2.5 to 3.3 kg medium fine wool, and have a mature liveweight of 23 to 45 kg.

### Animals and management

All procedures performed in studies involving animals were in accordance with the ethical standards of the Welfare and Ethics Committee of the Chinese Association for Laboratory Animal Sciences (SAC/TC 281). Sixty Pengbo semi-wool ewes, approximately 1.5 years of age with an average body weight of 31.33±0.43 kg, were randomly assigned to two treatment groups (n = 30 sheep in each), either the grazing (G) or the dry lot feeding (D) group. In summer and early autumn, all ewes were grazed on available grassland during the day and returned to separate individual sheds in the evening. During winter and spring, the ewes in the G group were grazed continuously each day and housed in one shed each evening over the experimental period. The ewes in the D group were housed in another shed each day and allowed access to a walled yard through the middle of the day. The structure and condition of the individual sheds in each group was the same. The sheds were adjacent and their floor areas were 40 m^2^ with a 60-m^2^ walled yard outside each shed, potential wind speed differences between the walled yard and the sheep grazing areas. Three troughs made of iron were placed in each walled yard allowed the ewes free access to supplements. The sheds had the inclined front sunlight-roof and gaps were filled with blankets to minimize airflow. A pair of windows on the front wall could be opened when appropriate to improve airflow. The average night temperatures inside the sheds of groups G and D were 2.03°C and 2.42°C, respectively.

All animals in each group were fed the same amount of commercial concentrate and *Secale cereale* grass hay. The commercial concentrate ration of 400 g/d per ewe was fed twice daily, at 9:00 and 19:00. Grass hay, salt licks, and water were available *ad libitum* to all ewes throughout the experimental period. The nutritive value of the commercial concentrate and grass hay is given in Table 1. All ewes were weighed once a month, prior to feeding or grazing in the morning.

### Air temperature recording

Temperatures in the shed were recorded at 30-min intervals with a recorder (JQA-1100DB, Qingdao Jiaqi Electron Equipment Co., Ltd., Qingdao, China) which was located in the middle of each roof beam. Temperatures in the field in which animals in the G group were grazed daily was recorded hourly using a HOBO weather station logger (H21, Onset Computer Corp., Bourne, MA, USA).

### Blood sample preparation

Blood samples were collected on the last day of the experiment, prior to the morning feed. The samples were taken from the jugular vein of six ewes in each group. For each ewe, 5 mL of blood was collected for evaluation of physiological and biochemical parameters. In addition, the serum was separated from 10 mL blood centrifuged at 3,000 r/min for 15 min at room temperature. The samples were collected and aliquoted into 2-mL plastic vials, which were stored at −80°C for subsequent determination of antioxidant capacity and metabolites.

### Chemical analyses

The physiological profile of the blood samples, including white blood cells (WBC), red blood cells, hemoglobin, and platelets, was determined with an automated hematology analyzer (Celltac E MEK-7222K, Nihon Kohden, Tokyo, Japan); the biochemical profiles, including total protein, albumin, globulin, glucose, urea, cholesterol, triglyceride, high density lipoprotein-cholesterol (HDL-C), and low density lipoprotein cholesterol were determined using an automatic biochemistry analyzer (Hitachi 7020, Hitachi High Technologies, Inc., Ibaraki, Japan). Serum antioxidant capacity, including total antioxidant capacity (T-AOC), superoxide dismutase (SOD), glutathione peroxidase (GSH-Px) and malondialdehyde (MDA) activity, was tested using a commercial biochemical reagent kit (Nanjing Jiancheng Bioengineering Institute, Nanjing, China) according to the manufacturer’s instructions.

### Metabolomics analysis

#### Non-targeted metabolomics analysis

All serum samples were acquired by the liquid chromatography mass spectrometry (LC-MS) system following the machine instructions. First, all chromatographic separations were performed using an ultra performance liquid chromatography (UPLC) system (Waters, Elstree, UK). An ACQUITY UPLC BEH C18 column (100 mm×2.1 mm, 1.7 μm, Waters, UK) was used for the reversed phase separation. The column oven was maintained at 50°C. The flow rate was 0.4 mL/min and the mobile phase consisted of solvent A (water+0.1% formic acid) and solvent B (acetonitrile+0.1% formic acid). Gradient elution conditions were set as follows: 0 to 2 min, 100% phase A; 2 to 11 min, 0 to 100% B; 11 to 13 min, 100% B; 13 to 15 min, 0 to 100% A. The injection volume for each sample was 10 μL.

A high-resolution tandem mass spectrometer, Xevo G2 XS QTOF (Waters, UK), was used to detect metabolites eluted from the column. The QTOF was operated in both positive and negative ion modes. For positive ion mode, the capillary and sampling cone voltages were set at 3 kV and 40 V, respectively. For negative ion mode, the capillary and sampling cone voltages were set at 1 kV and 40 V, respectively. The mass spectrometry data were acquired in Centroid MSE mode. The time-of-flight mass range was from 50 to 1,200 Da and the scan time was 0.2 s. For the MS/MS detection, all precursors were fragmented using 20 to 40 eV, and the scan time was 0.2 s. During the acquisition, the LE signal was acquired every 3 s to calibrate the mass accuracy. Furthermore, in order to evaluate the stability of the LC-MS during the whole acquisition, a quality control sample (pool of all samples) was acquired after every 10 samples.

#### Metabolic profiling and metabolite analysis

The raw data acquired were analyzed using Progenesis QI v2.2 software (Nonlinear Dynamics, Newcastle, UK) and metaX as an R package for peak detection and alignment. The intensity of each ion was normalized with regard to the total ions, using the count to generate a data matrix that consisted of the retention time, m/z value, and the normalized peak area. The retention time and m/z data for each peak were determined by the aforementioned software. Further analyses of the data matrix were performed using SIMCAP+software 12.0 (Umetrix AB, Umea, Sweden) for the principal component analysis (PCA) and partial least square discriminant analysis (PLS-DA).

Identified metabolites were further functionally and metabolically characterized using the Kyoto encyclopedia of genes and genomes (KEGG) and the human metabolome database (HMDB) database. Furthermore, the construction, interaction and pathway analysis of potential biomarkers was performed with software based on database sources, including the KEGG and HMDB, to identify the metabolic pathways.

### Statistical analysis

The data on the monthly changes in the temperature in the field and each treatment during December to April were analyzed using repeated measures in the MIXED procedure of SAS, with a model that included fixed and interactive effects of treatment and month. When the factors were significant (p<0.05), means were compared using Tukey’s test. The body weight, as well as physiological and biochemical parameters and antioxidant capacity in the blood of the ewes, were examined using a two-sample t-test for means with a model that included treatment effects and experimental error, and the results were expressed as mean±standard error. Differences were considered significant when p<0.05.

For serum metabolites, PCA, an unsupervised method, was used to find the direction of maximum variance in a complex collection of data. PLS-DA, a supervised method, was used to perform classification and feature selection. Furthermore, a variable important for the projection score was used to rank the metabolites on the basis of their importance in discriminating treatment groups. To assess the statistical significance of class discrimination in the PLS-DA model, a permutation test was performed. In addition, a one-way analysis of variance (ANOVA) with Tukey’s honestly significant difference (HSD) test was performed on the metabolomics data, in order to assess which metabolites were mainly involved in each of the various groups. The threshold of significance was set at p<0.05.

## RESULTS

### Differences in ambient temperatures in the field and sheepfold

**Figure 1:**
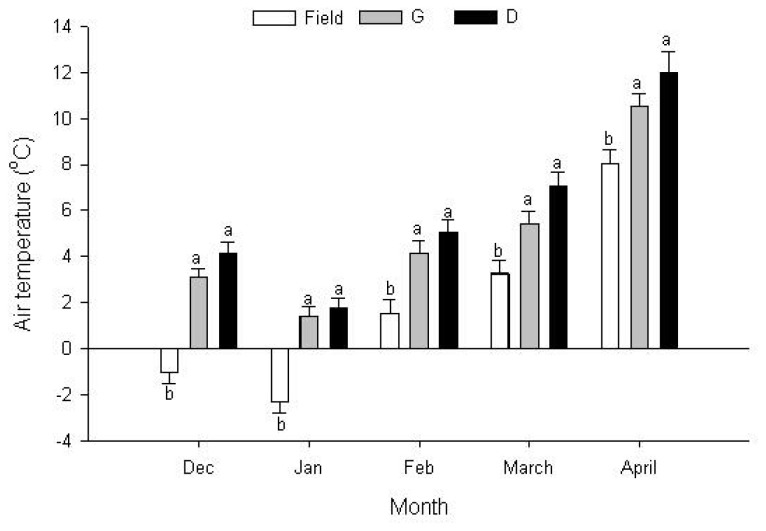
Changes in air temperatures in field, grazing sheepfold (G) and indoor feeding sheepfold (D) in each month from December to April. The data used were monthly mean values of the three conditions. Significant differences (p<0.05) are represented by different letters. Error bars are a standard error.

There were significant differences in air temperature between the field and sheep sheds (Figure 1). The average temperature value from December to April was higher (p<0.001) inside the two sheds (4.92°C and 6.0°C) than that in the outside field (1.89°C), but no significant difference was observed between the two sheds.

### Differences in the weight gain of ewes

The live weight and weight gain of ewes were significantly affected by treatment (Table 2). The final weight was 28.8 kg in the G group, compared with 32.4 kg in the D group (p = 0.020). Consequently, the weight gain of the G group (−22.8 g/d) was significantly lower (p = 0.011) than that of the D group (5.59 g/d).

### Differences in the blood physiological and biochemical parameters of ewes

Most of the physiological parameters did not significantly differ according to the treatment, but the number of WBCs and platelets were higher in the D group than in the G group (p = 0.037, p = 0.023; Table 3), whereas the ewes in the G group had a higher platelet-large cell ratio than the D group ewes (p = 0.028). The values of cholesterol and HDL-C were lower in the D group than in the G group (p = 0.039; p = 0.008), and there were no significant differences in other biochemical parameters between the groups.

### Differences in the serum antioxidant capacity of the ewes

The antioxidant capacity was measured in serum samples from the ewes given different treatments. The ewes in the G group had higher GSH-Px activity compared with the ewes in the D group (p = 0.041; Table 4), but there was no significant difference in T-AOC, SOD, and MDA values between the groups.

### Differences in serum metabolites of the ewes

**Figure 2:**
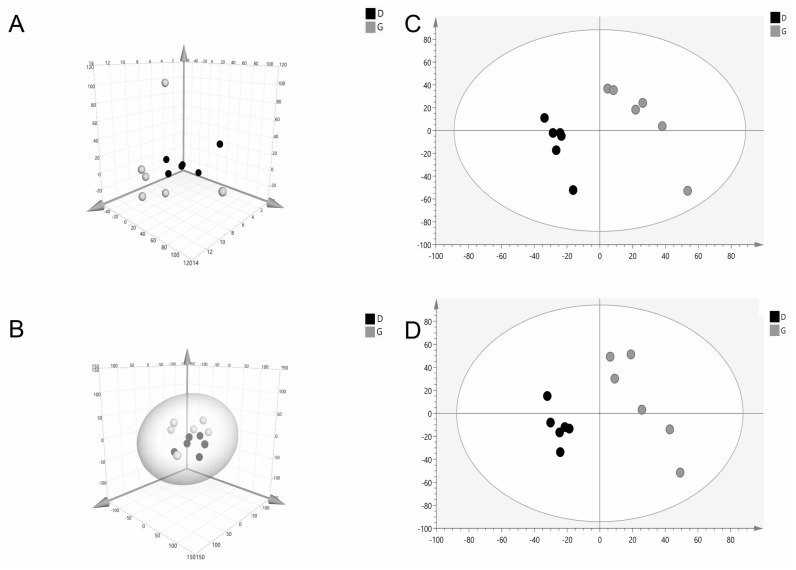
Multivariate data analyses of the LC/MS serum spectra data. 3D plot of principal component analysis of the G group compared with the D group in positive mode (A); and negative mode (B); partial least square discriminant analysis (PLS-DA) S-plot for in positive mode (C); and negative mode (D). Gray plot represents G group, the grazing group. Black plot represents D group, the drylot feeding group. A separation between G and D groups is observable for the investigated analytical conditions.

**Figure 3:**
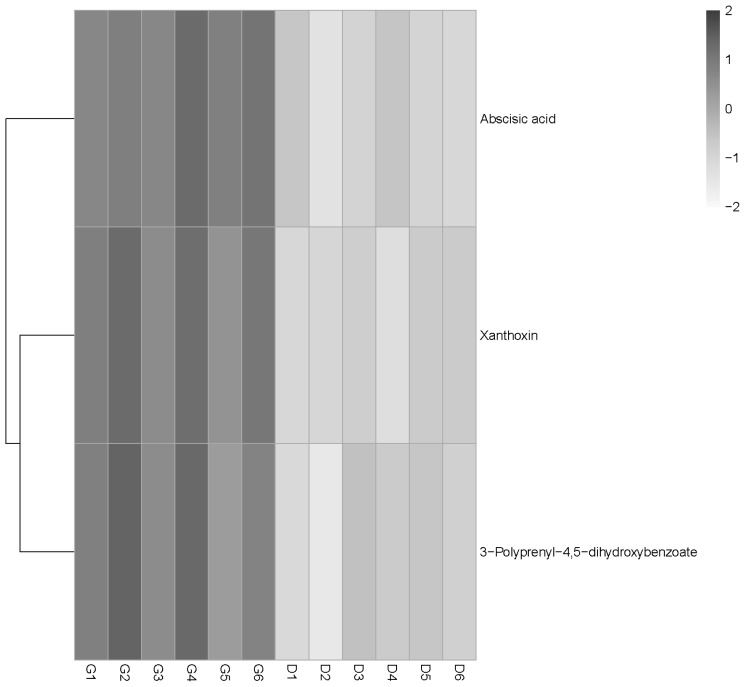
The heat map depicted here with black band indicates an increase in metabolite level of the grazing group (G) (fold change <1.2) and the gray band indicates a decrease in metabolite level of the drylot feeding group (D) (fold change >1.2). Each row represents a metabolite and each column represents a sheep sample. Different colors represent different intensities, from gray to black, represents low to high of metabolite intensities.

The PCA score scatter plot is shown in Figure 2. The PCA 3D score plot showed that the metabolic profiles in the G and D groups did not have a clear separation (Figure 2A, 2B). The supervised analysis, PLS-DA, was subsequently performed to maximize the separation and identify those metabolites contributing to the observed separation. In the PLS-DA score plot (Figure 2C, 2D), the separation between the G and D groups was more prominent. In order to confirm the specificity and the significance of important metabolites identified from PCA and PLS-DA, we performed univariate analysis using one-way ANOVA and Tukey’s HSD test on each metabolite. Thirteen metabolites were significantly different between the groups (p≤0.05). In addition, a total of three potential metabolites, abscisic acid (ABA), xanthoxin and 3,4-dihydroxy-5-polyprenylbenzoate (DHHB), were selected for further investigation. Interestingly, all these compounds were upregulated (p<0.05) in the G group and acted to disturb metabolic pathways (Figure 3, Table 5).

## DISCUSSION

### Effects of indoor housing on ewe liveweight

Low winter temperatures and chilling winds cause cold stress by increasing the energy requirements of livestock, leading to large decreases in productivity. For adult sheep, the lower critical temperature is −3°C [8], below which daily metabolisable energy requirements increase by 0.14 to 0.64 MJ for each 1°C decrease in ambient temperature from January to December (60-kg sheep) [9]. Our previous study has found that the appropriate ambient temperature for adult ewes in northwest pastoral regions of China should be above 2°C [10]. In Linzhou County in Tibet, where high altitude, low winter temperatures and wind chill factors result in high energy demands, the animals will lose weight. During the entire experimental period, the average temperature inside the two sheds did not differ significantly, but average temperature outdoors in winter (December to February) was −0.61°C, well below the indoor temperature (3.65°C) and the range reported by Zhang et al [10]. Such low winter temperatures led to a large weight loss (nearly 23 g/d) in the ewes that were grazed daily, which means that considerable amounts of energy from high-quality supplements would be needed to prevent any weight loss and maintain body temperature. As estimated by Young [11], feed requirements for overwintering beef cows are elevated by 30% to 70% under adverse winter conditions. However, in the present trial, the ewes in the G and D groups were fed the same quantity of concentrate (400 g/d), and the ewes grazed outdoors still lost weight at 22.8 g/d although medium quality grass hay (with 71.9 g/kg DM crude protein and 706 g/kg DM neutral detergent fiber, respectively) was supplied freely during the experimental period. This is mainly attributed to the harsh wind and low temperature in winter–sping in the local area. During the coldest month, the wind speed is about 15 km/h (= 4.2 m/s) and the outdoor temperature averages −2.29°C, which resulted in remarkable weight loss (22.8 g/d) by the ewes grazed outside daily. However, housing in a mild shed without grazing can protect sheep from acute cold during winter [12] and reduce their weight loss [4]. As the report by Tuo et al [13] found, yaks (6 to 8 years of age) fed in a warm shed had a higher live weight than yaks grazing (+283 g/d), and the daily weight gain of the warm-shed yak calves was 189 g/d greater than that of calves from grazing yaks. Similar findings were observed for the marketing rate of Tibetan sheep and yaks by Zhen et al [6], who reported that the marketing rate for Tibetan sheep and yaks, respectively, increased by 3% to 9% and 2% to 3% following implementation of a crop-forage regime plus warm sheds. Additionally, the warm sheds also resulted in more animals to rear and a greater capability to avoid mortality, especially for young animals and adult females, which led to higher incomes.

In the present study, the ewes allocated to the indoor feeding maintained significantly higher live weight and increased weight gain by 125%, as compared with the ewes kept grazing outdoors (5.59 vs −22.8 g/d). This is because the indoor feeding reduced the long-term cold stress during daily grazing as well as reducing exposure to the harsh wind chill. The benefits of indoor feeding may also be related to the reduced energy requirements due to walking activity and the low quality forage available to the sheep. Therefore, local herders should focus more on housing sheep indoors, preferably in a warm shed, and feeding animals rather than grazing them outdoors during winter–spring, which would decrease the weight loss of ewes, and allow better management of degraded grasslands.

### Effects of indoor housing on blood physiological and biochemical profiles in the ewes

Hematological examination reflects an animal’s responses to its external and internal environments. The normal values of physiological and biochemical indexes are used for evaluating stress and the well-being of an animal. In the present study, winter grazing of ewes resulted in a decreased WBC count, in line with the report by Zakari et al [14], who found that the WBCs of donkeys decreased during the cold dry season. The physiological responses of animals to environmental stress and their energy balance have profound effects on serum biochemical parameters. Soon after the beginning of stress or during mild short-term stress, stress hormones, such as glucocorticoids, induce the release of leukocytes from organs, and these enter the blood vessels and lymphatics [15]. This is a consequence of increased blood leukocyte numbers. As the stress response continues, stress hormones induce leukocytes to exit the blood and redistribute in the lungs, liver and lymph nodes in preparation for immune challenges [16], which results in a decrease in blood leukocyte numbers [17]. Low temperature was one of the most serious threats for the winter grazing sheep used in this trial. The long-term (5 months) cold grazing induced a cold stress response, resulting in a decrease in WBCs in the grazing sheep (G group). It is important to note, however, that the WBC values were above the reference range for animals in other regions in China [18]. This effect is highly likely to be attributable to the adaptation of these animals to the high altitude conditions in Tibet.

Compelling evidence suggests that platelets may act as one of the regulators of the immune response by activating the immune system during autoimmune processes [19]. In this study, all platelet parameters, in both the G and the D sheep, were within the reference range [20]. However, lower numbers of platelets were observed in ewes grazed outdoors, which is in line with the finding of Zhao et al [21], who found that the number of platelets in Holstein cattle decreased from 279 ×10^9^ to 60.8×10^9^/L when the temperature decreased from −5°C to −20°C.

Moreover, the serum metabolomic data showed that the grazing ewes had higher ABA and xanthoxin concentrations than indoor housed ewes. Xanthoxin is a natural precursor of ABA [22], which is a 15-C weak acid involved in the response to environmental stress in both plants and animals [23]. ABA can be produced and released from many animal and human cells (immune cells, cardiovascular cells, and pancreatic cells) under physiological or pathological conditions, and regulates cell growth, development and immune responses to various stimuli through a signaling pathway [24].

From the above findings regarding the physiological profile, it can be deduced that the grazing sheep exposed to long-term cold stress showed induction of immunoprotective responses through changing levels of WBCs and platelets, as well as serum metabolites. This long-term stressor may reduce the health of ewes.

As demonstrated by Zhang et al [4], cold grazing in winter leads to high levels of heat loss from ewes and serious energy imbalance. The ewes in G group suffered severe cold stress during daily grazing, and the higher serum cholesterol and HDL-C concentrations were probably due to the thermogenesis used by the sheep to adapt to the cold environment. Cholesterol is synthesized as a response to energy metabolism in the liver. There is some evidence that cholesterol and total lipid concentrations were higher when fat-tailed sheep are reared in cold stress conditions [25]. Calves exposed to cold environments had an increase in blood lipid concentrations when compared with calves exposed to warm environments [26]. These findings further suggested the involvement of blood lipids in cold thermogenesis. HDL-C transports cholesterol from peripheral cells to the liver for recycling and disposal [27], and evidence suggests that exercise will be effective in increasing HDL-C [28]. The ewes used in this trial that grazed outdoors daily experienced long durations and long distances of walking exercise every day and this may have increased the concentration of HDL-C.

### Effects of indoor housing on the serum antioxidant capacity of ewes

Animals have evolved highly complex antioxidant systems (enzymic and nonenzymic), which work synergistically to protect the cells and organ systems of the body against free radical damage. The most efficient enzymatic antioxidant systems involve SOD and GSH-Px. As expected, indoor housing resulted in decreased GSH-Px activity in the ewes in the current study. GSH-Px contains Se, which is tightly bound, and its activity in various tissues from several species was found to be dependent on dietary Se intake [29]. In this study, the grazing ewes had higher GSH-Px activity, which may be ascribed to their intake of diverse herbage compounds, compared with the single simple forage source provided to the housed ewes. The lack of differences in serum T-AOC and MDA concentrations between the groups implied that the winter housing feeding system in this trial may not have been sufficient to influence the total antioxidant ability and lipid peroxidation.

The results from the metabolomics analysis showed that the sheep fed indoors had decreased DHHB, as compared with those grazed outdoors. DHHB is an intermediate that participates in the biosynthesis pathway of ubiquinone and plays a pivotal role in inner mitochondrial membrane electron transport; it also serves as an important endogenous antioxidant, preventing lipid peroxidation in cell membranes [30]. The higher serum DHHB concentration in the G group reflects the increased energy production and better cell antioxidant activity, which is consistent with the above results. It suggests that grazing sheep can supply the extra energy consumption required by long distance walking, accompanied by the chill and wind in the cold season, by thermogenesis through increasing lipid metabolism. At the same time, along with the enhancement of mitochondrial energy metabolism, free radical production also increased. Therefore, the grazing ewes showed increased ubiquinone content and GSH-Px activity as a compensatory antioxidant mechanism.

Nevertheless, the temperatures both inside and outside the sheds were below the optimum temperature range for sheep in the cold season, leading to low efficiency in the growth performance of local sheep, even when given the same supplementation. In this study, low numbers of metabolites in serum were tested, and the nutrient metabolites did not show differences. Therefore, the differences in serum metabolites in two groups were focused on the stress reaction.

## CONCLUSION

In Tibet, keeping livestock in sheds in winter–spring, in combination with feeding a moderate (or high) quantity of fodder, is a more efficient management strategy than daily grazing through winter that will enhance animal performance, leading to increased farmer incomes. Most importantly, winter–spring housing will reduce the cold stress on grazing animals, enhancing the blood physiological and biochemical profiles, and thereby possibly contribute to reduced management costs.

## Figures and Tables

**Table 1:** Chemical composition values of individual feeds on dry matter basis

Feed	Chemical composition				

	DM (g/kg)	CP (g/kg)	ME (MJ/kg)	NDF (g/kg)	ADF (g/kg)
Concentrate1)	921.7	161.2	11.2	96.8	60.1
*Secale cereale* hay	935.4	71.9	7.25	696.1	392.0

DM, dry matter; CP, crude protein; ME, metabolisable energy; NDF, neutral detergent fiber; ADF, acid detergent fiber.

1) Concentrate consisted of chopped maize 65%, soybean meal 10%, cottonseed meal 8%, rapeseed meal 6%, wheat bran 10% and premix 1%.

**Table 2:** Changes in liveweight of ewes grazed outside (G) and fed indoors (D)

Item	Treatment		p-value

	G	D	
Starting weight (kg)	31.2±0.5	31.5±0.3	0.858
Final weight (kg)	28.8±1.2*	32.4±0.9*	0.020
Weight gain (g/d)	−22.8±7.8*	5.59±7.36*	0.011

* Means within each row differ significantly (p<0.05).

**Table 3:** Physiological and biochemical parameter in blood of ewes grazed outside (G) and fed indoors (D)

Item	Treatment		p-value

	G	D	
Physiological parameters			
White blood cell (×10^9^/L)	138.5±7.2*	159.3±5.4*	0.037
Hemoglobin (g/L)	128.2±10.9	135.5±4.3	0.441
Red blood cell (×10^12^/L)	8.35±0.72	8.40±0.33	0.882
Platelet (×10^9^/L)	192.8±72.4*	379.0±53.2*	0.023
Mean platelet volume (fL)	6.07±0.49	5.70±0.50	0.229
Platelet distribution width (%)	14.7±0.2	14.6±0.1	0.893
Plateletcrit (%)	0.13±0.03	0.17±0.02	0.211
Platelet-large cell ratio (%)	13.1±3.5*	7.8±1.7*	0.028
Biochemical parameters			
Total protein (g/L)	72.6±5.7	70.4±4.4	0.472
Albumin (g/L)	25.3±1.2	24.4±1.4	0.187
Globulin (g/L)	49.2±4.1	48.0±5.5	0.693
Glucose (mmol/L)	3.25±0.37	3.02±0.52	0.418
Urea (mmol/L)	8.74±1.13	7.52±1.23	0.105
Cholesterol (mmol/L)	1.66±0.26*	1.36±0.18*	0.039
Triglyceride (mmol/L)	0.31±0.13	0.29±0.16	0.731
High-density lipoprotein cholesterol (g/mL)	1.40±0.05*	1.29±0.06*	0.008
Low density lipoprotein cholesterol (g/mL)	0.40±0.10	0.34±0.08	0.381

* Means within each row differ significantly (p<0.05).

**Table 4:** Serum antioxidant capacity of ewes grazed outside (G) and fed indoors (D)

Item	Treatment		p-value

	G	D	
Total antioxidant capacity (nmol/mg prot)	3.72±0.20	3.54±0.15	0.485
Superoxide dismutase (U/mg prot)	117.9±4.2	110.2±3.7	0.202
Glutathione peroxidase (nmol/mg prot)	123.4±8.9*	97.8±5.2*	0.041
Malondialdehyde (nmol/mg prot)	4.26±0.30	3.85±0.20	0.138

* Means within each row differ significantly (p<0.05).

**Table 5:** Identification and trends of change for differential metabolites

Metabolite	Formula	p-value	VIP	Retained time	m/z	Trend	Metabolic pathway
Abscisic acid	C_15_H_20_O_4_	0.004*	6.99	4.55	247	↑	Metabolic pathways
Xanthoxin	C_15_H_22_O_3_	0.008*	5.96	5.37	233	↑	Metabolic pathways
3-Polyprenyl-4,5-dihydroxybenzoate	C_12_H_14_O_4_·[C_5_H_8_]n	0.044*	5.11	5.37	291	↑	Ubiquinone and other terpenoid-quinone biosynthesis

VIP, variable important for the projection.

* Means within each row differ significantly (p<0.05).
